# Integrated 16s RNA sequencing and network pharmacology to explore the effects of polyphenol-rich raspberry leaf extract on weight control

**DOI:** 10.3389/fnut.2023.1306037

**Published:** 2024-01-08

**Authors:** Tao Wang, Jing Yang, Ziang Huang, Fei Wang, Ruzi Liu, Yongping Liu, Xiaojun Li

**Affiliations:** ^1^School of Chemistry and Chemical Engineering, North University of China, Taiyuan, Shanxi, China; ^2^Dezhou Industrial Technology Research Institute of North University of China, Dezhou, Shandong, China; ^3^The Hospital of North University of China, Taiyuan, Shanxi, China; ^4^Dezhou Yongshengzhai Braised Chicken Group Co., Ltd., Dezhou, Shangdong, China

**Keywords:** obesity, *Rubus idaeus*, intestinal flora, network pharmacology, IL-6, TNF-α

## Abstract

**Introduction:**

Obesity is recognized as a chronic low-grade inflammation associated with intestinal flora imbalance, leading to dyslipidemia and inflammation. Modern research has found that polyphenols have anti-obesity effects. However, the mechanism of action of raspberry leaf extract (RLE) with high polyphenols in regulating obesity is still unknown. This study investigated the improvement effect of supplementing RLE on high-fat diet (HFD) induced obesity in mice.

**Methods:**

RLE was used to intervene in HFD induced C57BL/6J male mice during prevention stage (1-16 weeks) and treatment stage (17-20 weeks). Their weight changes and obesity-related biochemical indicators were measured. The changes in intestinal flora were analyzed using 16S rRNA sequencing, and finally the targets and pathways of the 7 typical polyphenols (quercetin-3-O-glucuronide, ellagic acid, kaempferol-3-O-rutinoside, chlorogenic acid, brevifolin carboxylic acid, quercetin-3-O-rutinoside, and quercetin) of RLE in the regulation of obesity were predicted by network pharmacology approach.

**Results and discussion:**

The results showed that RLE effectively prevented and treated weight gain in obese mice induced by HFD, alleviated adipocyte hypertrophy, reduced Interleukin-6 and Tumor Necrosis Factor Alpha levels, and improved intestinal flora, especially *Muriaculaceae*, *Alistipes* and *Alloprevotella*, and decreased the *Firmicutes/Bacteroidota* ratio. Network pharmacology analysis selected 60 common targets for 7 RLE polyphenols and obesity. Combined with protein-protein interaction network, enrichment analysis and experimental results, TNF, IL-6, AKT1, and PPAR were predicted as potential key targets for RLE polyphenols.

**Conclusion:**

The potential mechanism by which polyphenol-rich RLE regulates obesity may be attributed to the specific polyphenols of RLE and their synergistic effects, therefore RLE has a great anti-obesity potential and may be used as a means to alleviate obesity and related diseases.

## Introduction

The World Health Organization (WHO) defines both overweight (a body mass index (BMI) ≥ 25 kg/m^2^) and obesity (BMI ≥ 30 kg/m^2^) as an abnormal or excessive fat accumulation (1). Obesity greatly increases the risk of diseases including type 2 diabetes, fatty liver, hyperglycemia and coronary heart disease, as well as reduces quality of life and life expectancy (2, 3). Since 1980, the frequency of overweight and obese individuals has doubled, hence, obesity has become a growing public health problem (1). Although changing lifestyle, adjusting diet, and increasing physical activity should be the best choices for controlling weight, overweight or obese individuals, even lean individuals may prefer to diet pills. Orlistat, naltrexone/bupropion and liraglutide are commonly weight-loss pills that have received approval in the USA or European Union (4). Unfortunately, orlistat commonly causes increased bloating, and stomach pain (5), while liraglutide is a glucagon-like peptide-1 agonist, and the most common side effect is nausea, and gastrointestinal disorders such as constipation and diarrhea (6). Therefore, safer and more effective methods should be sought for treating obesity.

Increasing evidence suggests that consuming a diet high in polyphenols may be beneficial for antioxidation and anti-obesity (7). The raspberry (*Rubus idaeus* L.), or Fu Pen Zi in Chinese, is a *Rubus* plant in the Rosaceae family (8). Unripe raspberry (*Rubus Chingii* Hu) is a typical Traditional Chinese Medicine (TCM) (9), rich in ellagic acid, flavanols, flavonols, phenolic acids and so on (10, 11). Among them, chingiitannin A exhibits the best inhibitory activity against α- amylase and α- glucosidase (12). In Europe and the United States, raspberry is known as the “Fruit of Life.” According to the data from the Food and Agriculture Organization of the United Nations (FAO), it is the third largest high-value small berry in the world, with an annual demand of more than 2 million tons (13). Raspberry leaves are also rich in polyphenols, ellagic acid, quercetin and their derivates (14). They were included in Chinese Mmedicine and Food Homologous List in 2002 (15), the Chinese Cosmetic Raw Materials list in 2015 (16), the Scientific Committee of the British Herbal Medicine Association in 1983 (17), and the European Community Herbal Medicine Monograph published by the European Medicines Agency (EMA) in 2012 (18). In our previous studies, it was shown that the total phenolic content (TPC) of raspberry leaf extract (RLE) reached 540.32 ± 8.96 mg g^−1^, and RLE supplementation reduced body weight and adipose percentage in mice after ingestion (19). In addition, the 16 s RNA sequencing results of co-cultivation of RLE with volunteer feces showed that RLE has potential value in maintaining intestinal health (20).

Dysbiosis of the intestinal flora is a common symptom of obesity and other metabolic diseases (21). It has been found that obese mice have more *Firmicutes* and significantly fewer *Bacteroidetes* than lean mice (22). The ratio of *Firmicutes*/*Bacteroidetes* (*F/B*) is often used as a marker of obesity, with the ratio being greater in obese animals (23) and in obese subjects (24). In the study of the effects of polyphenols on intestinal flora, supplementation with anthocyanin component and metabolite of Saskatoon berry increased the abundance of *Muribaculaceae* (25), as well as supplementation with polyphenol-rich oolong tea increased the abundance of bacteria (*Bacteroides*, *Alistipes*, and *Alloprevotella*) that can produce short chain fatty acid (SCFA) (26). These polyphenol supplements show great value in improving the intestinal flora.

Traditional herbs and herbal extracts have complex compositions and usually act on multiple targets to achieve therapeutic effects on diseases (27). The use of network pharmacology analysis can greatly reduce the initial screening cost and systematically explain the therapeutic mechanism of herbal medicines on diseases (28). For example, Wang et al. (29) reported that the anti-obesity mechanism of baicalein may be related to the up-regulation of SLC2A1 and the down-regulation of TNF by network pharmacology prediction and experimental validation. Li et al. (30) also studied the obesity-relieving mechanism of mulberry (*Morus alba* L.) leaves and screened the potential anti-obesity mechanism using network pharmacology, which was verified through experiments. The results showed that the anti-obesity mechanism of mulberry leaves involved inflammation, lipid metabolism, and PI3K/Akt/Bcl-xl signaling pathway.

The purpose of this study is to further investigate the effects of RLE on obese C57BL/6J male mice induced by high-fat diet (HFD). RLE supplementation was conducted during the prevention and treatment stages of obese mice, respectively. The body weight, blood glucose levels, blood lipids, and inflammatory factors of mice were measured, their fat and liver cells were observed, as well as 16 s RNA intestinal flora were sequencing. Meantime, the targets and pathways of 7 typical polyphenols in RLE in obesity regulation were predicted through network pharmacology. The results will further explore the intrinsic mechanism of RLE and its main compounds in controlling body weight.

## Materials and methods

### Chemicals

Basic diet (12% of energy from fat in the basal diet SWS9102) was provided by Jiangsu Synergetic Pharmaceutical Bioengineering Co., Ltd. (Jiangsu, China). Interleukin-6 (IL-6) and Tumor Necrosis Factor Alpha (TNF-α) commercial reagent kit were provided by Wuhan Purity Bioengineering Co., Ltd. (Wuhan, China). TIANamp Stool NDA Kit was provided by Tiangen Bioengineering Co., Ltd. (Beijing, China). Paraformaldehyde (4%), hematoxylin, eosin and other reagents were provided by Shanghai yuanye Bioengineering Co., Ltd. (Shanghai, China). Raspberry leaf extract (RLE) with a total phenolic content (TPC) exceeding 50% was prepared in the laboratory according by Yang et al. (19).

### Animal experiments

**Figure 1:**
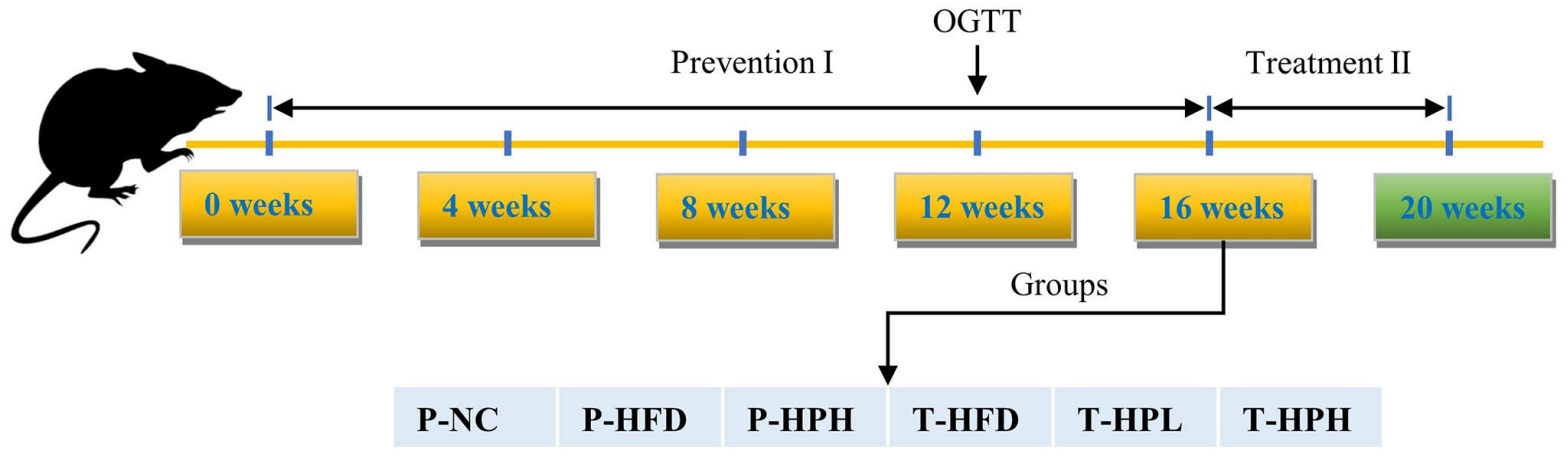
Schematic diagram of mice grouping.

Thirty 4-week-old specific pathogen free C57BL/6 J male mice were purchased from Beijing Vital River Laboratory Animal Technology Co., Ltd. (Beijing, China). All mice were housed under controlled conditions of 23 ± 2°C and a 12 h light–dark cycle. The grouping diagram and experimental process were as follows Figure 1 and Table 1, and the entire experiment period lasted for 20 weeks. After one week of adaptation, the mice were randomly divided into three groups: P-NC (*n* = 6, basal diet SWS9102 with 12% energy), P-HFD (P-HFD group, *n* = 18, HFD: SWS9102 + 19.2% lard with 45% energy), and P-HPH (P-HPH group, *n* = 6, HFD with 0.2% RLE). After 16 weeks, the remaining mice in the P-HFD group were randomly divided into three groups: T-HFD (T-HFD group, *n* = 4, HFD), T-HPL (T-HPL group, *n* = 4, HFD with 0.1% RLE), and T-HPH (T-HPH group, *n* = 4, HFD with 0.2% RLE). All animal experimental procedures were in accordance with the requirements of the China National Standard for Laboratory Animals. Mouse body weight was recorded after fasting for 12 h each week. At the end of each stage, mice were euthanized by cervical dislocation after blood collection from the orbital venous plexus, and white adipose tissue, liver and rectal feces collections for subsequent experiments.

**Table 1:** Mice grouping and food.

Group	P-NC	P-HFD	P-HPH	T-HFD	T-HPL	T-HPH
Stage	Prevention I (1–16 weeks)			Treatment II (17–20 weeks)		
No.	*n* = 6	*n* = 18	*n* = 6	*n* = 4	*n* = 4	*n* = 4
Food	Normal control diet	High-fat diet				
Fat energy supply ratio	12% (SWS9102)	45% (SWS9102 + 19.2% lard)				
RLE	0	0	0.2%	0	0.1%	0.2%

### Serum biochemical analysis

After fasting for 12 h, blood samples were obtained from mice through the orbital venous plexus. The samples were stored overnight at 4°C and then centrifuged at 3000 rpm for 15 min. The supernatant serum was transferred and stored at −20°C for future use. The contents of total cholesterol (TC), total triglycerides (TG), low-density lipoprotein cholesterol (LDL-C), and high-density lipoprotein cholesterol (HDL-C) in the serum were measured using 7,180 Full-automatic biochemical analyzer (Hitachi High-Tech Diagnostics Co., Ltd., Shanghai, China).

### Oral glucose tolerance test (OGTT)

After fasting for 16 h, a glucose solution of 2 g/kg body weight was gavaged to mice. Blood samples were collected by cutting off the tail tip at 0, 30, 60, 90, and 120 min after administration. The blood glucose levels were immediately measured using blood glucose meter (Jiangsu Yuyue Medical Equipment and Supply Co., Ltd., Jiangsu, China). The data of the OGTT were visualized using the area under the curve (AUC).

### Histopathological analysis

According to Dai’s method (31), fresh mouse liver and white adipose tissue were rapidly separated, fixed in 4% paraformaldehyde solution for 24 h. Then they were dehydrated in gradient alcohol, embedded in paraffin, sectioned, and stained with hematoxylin and eosin (H&E staining). After sealing with neutral gum, tissue pathological changes were observed by microscopy at 400 × magnification. The area of adipocytes was measured using Image J 2.0 software.

### 16s RNA sequencing

The intestinal microbial DNA of each group of mice was extracted using TIANamp Stool NDA Kit. A pair of specific primers, 341F 5’-CTACGGGNGGCWGCAG-3′ and 805R 5’-GACTACHVGGGTATCTAATCC-3′, were used to amplify the V3-V4 region of the bacterial 16S rRNA gene through PCR. The raw data was sequenced on the Illumina NovaSeq PE250 platform by Shanghai Weihuan Bioengineering Co., Ltd. (Beijing, China). Trimmmomatic PE was used to trim low-quality bases from the original data (32). The analysis process of Qiime 2 (33) was used for filtering, deduplication, base correction, and removal of chimeric sequences. DADA2 (34) was employed to denoise the original data, which was equivalent to clustering at 100% similarity, and only removing and correcting low-quality sequences, identifying chimeras using an algorithm, and directly removing redundant sequences. The obtained ASV/OUT sequences were annotated to obtain corresponding species information and species-based abundance distribution. Visualization and analysis of data using R (Version 3.6.1).

### Collection of seven polyphenolic targets

The Traditional Chinese Medicine Systems Pharmacology Database (TCMSP)^1^ (35) and the Swiss Target Prediction database^2^ (36) were used to predict potential targets of 7 polyphenols (chlorogenic acid, brevifolin carboxylic acid, ellagic acid, quercetin-3-O-rutinoside, quercetin-3-O-glucuronide (Q3G), kaempferol-3-O-rutinoside (K3R), and quercetin). The target obtained from TCMSP were converted to *Homo sapiens* gene symbols using the UniProt database^3^ (37). The Swiss Target Prediction database was combined with the PubChem database^4^ (38). Canonical SMILES for 7 polyphenols were obtained from PubChem, and then they were imported into Swiss Target Prediction. The species selected “*Homo sapiens*,” and a screening condition of “probability” > 0 was used to screen for prediction of component action targets.

### Prediction of targets for obesity

Using “obesity” as a keyword, “Obesity”-associated target genes were predicted through the following 3 databases: GeneCards^5^ (39), Online Mendelian Inheritance in Man (OMIM)^6^ (40), and Therapeutic Target Database (TTD)^7^ (41). The overlapping targets between 7 polyphenol and obesity were considered as potential targets for obesity treatment. A Venn diagram was constructed using Jvenn^8^ (42) for visualization analysis.

### Protein–protein interaction analysis

The overlapping target were imported into the STRING database^9^ (43) to construct a protein–protein interaction (PPI) network. The network and enrichment facilities in STRING comprehensively characterized user gene lists and functional genomics datasets, and created and shared highly customized and enhanced protein–protein association networks (44). The species selected “*Homo sapiens*” and a minimum interaction score was set to the highest confidence (> 0.400). The PPI network data were imported into Cytoscape 3.9.0 software for visualization, and then the Cytoscape plugin centiscape 2.2 was used to calculate three specific centrality parameters of the PPI network topology structure. The parameters were key indicators for the nodes: “betweenness centrality (BC),” “closeness centrality (CC),” and “degree centrality (DC)” (45). Subsequently, using each parameter as a filtering condition, seven polyphenolic substances regulating obesity were selected as key targets. Finally, each parameter as a filtering condition screened key targets of 7 polyphenols for regulating obesity.

### Go and KEGG pathway enrichment analysis

The Metascape database^10^ (46) was used molecular function (MF) of Gene Ontology (GO), the enrichment of biological process (BP), and cellular component (CC), as well as the pathway analysis of Kyoto Encyclopedia of Genes and Genomes (KEGG). Then, the bubble charts and bar charts for data analysis and visualization were generated by online platform.^11^

### Statistical analysis

The data was represented as mean ± standard deviation (SD). Statistical analysis was conducted using SPSS 22.0 software. The data was evaluated through one-way analysis of variance (ANOVA) and then followed by Duncan’s multiple range test. *p* < 0.05 was defined as statistically significant.

## Results

### Effects of RLE supplementation on body weight in mice

**Figure 2:**
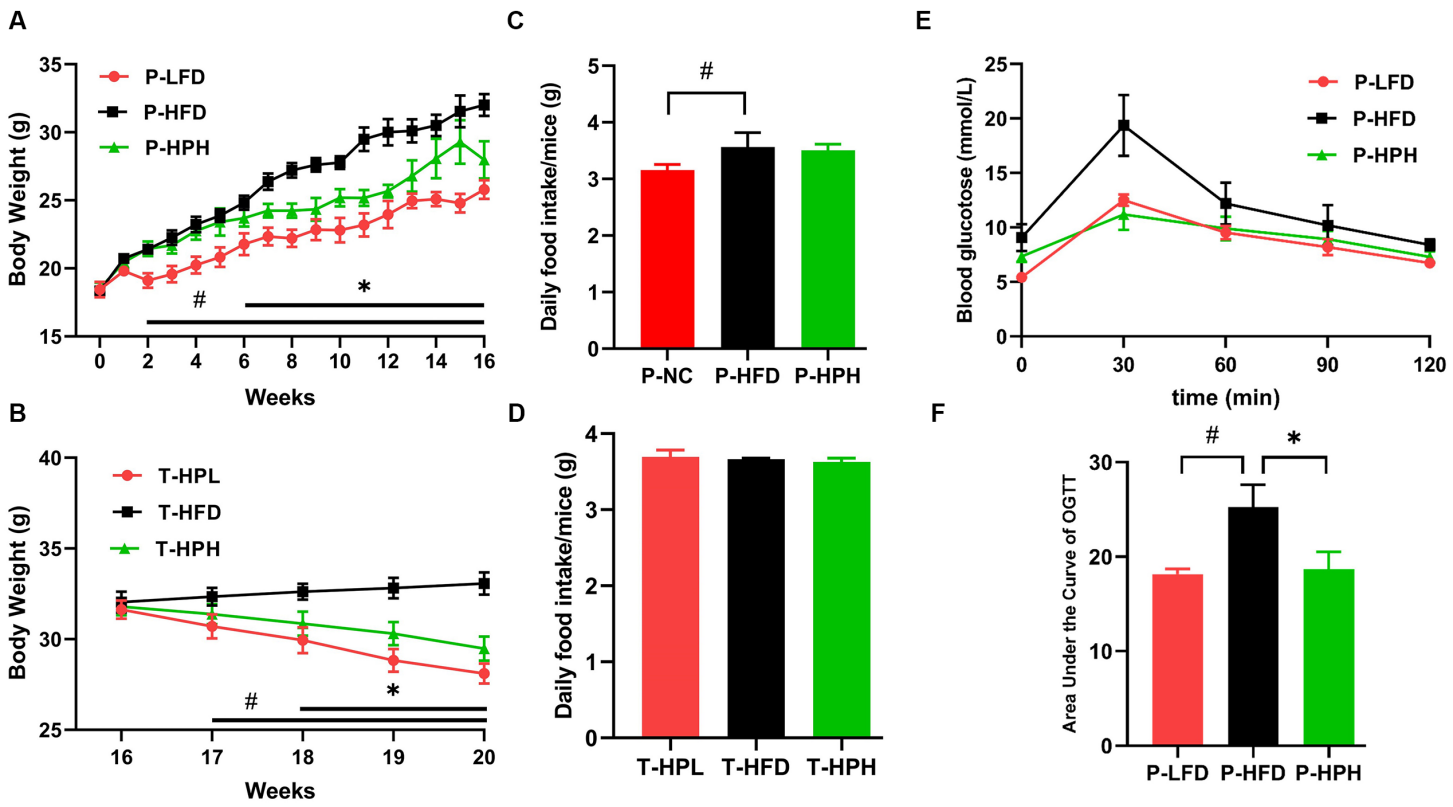
Effect of RLE in the prevention group on body weight and glucose tolerance in mice fed a high-fat diet. **(A,B)** Changes in body weight of mice. **(C,D)** Daily food intake. **(E)** OGTT. **(F)** AUC of OGTT. ^#^*p* < 0.05, P-HFD compared with P-LFD group. *p < 0.05, P-HFD compared with P-HPH group.

To investigate the effect of RLE on the body weight of mice fed HFD, the mice were divided into a prevention group (1–16 weeks) and a treatment group (17–20 weeks) at the 16-week mark (Figure 1; Table 1). As shown in Figure 2A, two weeks later, compared to the P-NC group, P-HFD group showed a significant increase in body weight (*p* < 0.05). Six weeks later, the weight of the P-HPH mice was significantly lower than that of the P-HFD group (*p* < 0.05). After 16 weeks, the weights of the P-NC, P-HFD, and P-HPH groups were 25.79 ± 0.69 g, 32.01 ± 0.80 g, and 28.97 ± 1.37 g, respectively. Compared with the P-NC group, the P-HPH group only increased by 12% in weight, while the P-HFD group increased by 24%. Subsequently, 12 mice with uniform body weight from P-HFD group were randomly divided into three groups (T-HFD, T-HPL, and T-HPH) for further treatment experiment. Their average weight was 31.82 ± 0.50 g, which was 23% heavier than the P-NC group. After 4 weeks, the body weights in the T-HPL group (28.11 ± 0.55 g) and the T-HPH group (29.48 ± 0.67 g) were significantly lower than those in the T-HFD group (33.07 ± 0.61 g) (*p* < 0.05) (Figure 2B). Meanwhile, there was no significant difference in food intake between RLE supplementation group and corresponding HFD group (Figures 2C,D). These results indicated that RLE supplementation could significantly reduce the weight gain induced by HFD in the preventive stage, and effectively reduced the body weight of obese mice in the treatment. These effects were not related to dietary intake.

### Effects of RLE on glucose tolerance and serum indexes

To investigate the effects of RLE on blood glucose regulation in obese mice, the OGTT test was implemented. As shown in Figure 2E, glucose absorption in the P-HFD group peaked at 30 min and then rapidly declined, while the P-HPH group showed slower absorption, with a similar overall trend to the P-NC group. At the AUC level (Figure 2F), a significant decrease was observed in the P-HPH group compared with the P-HFD group, suggesting that RLE ameliorated the glucose intolerance induced by HFD.

**Figure 3:**
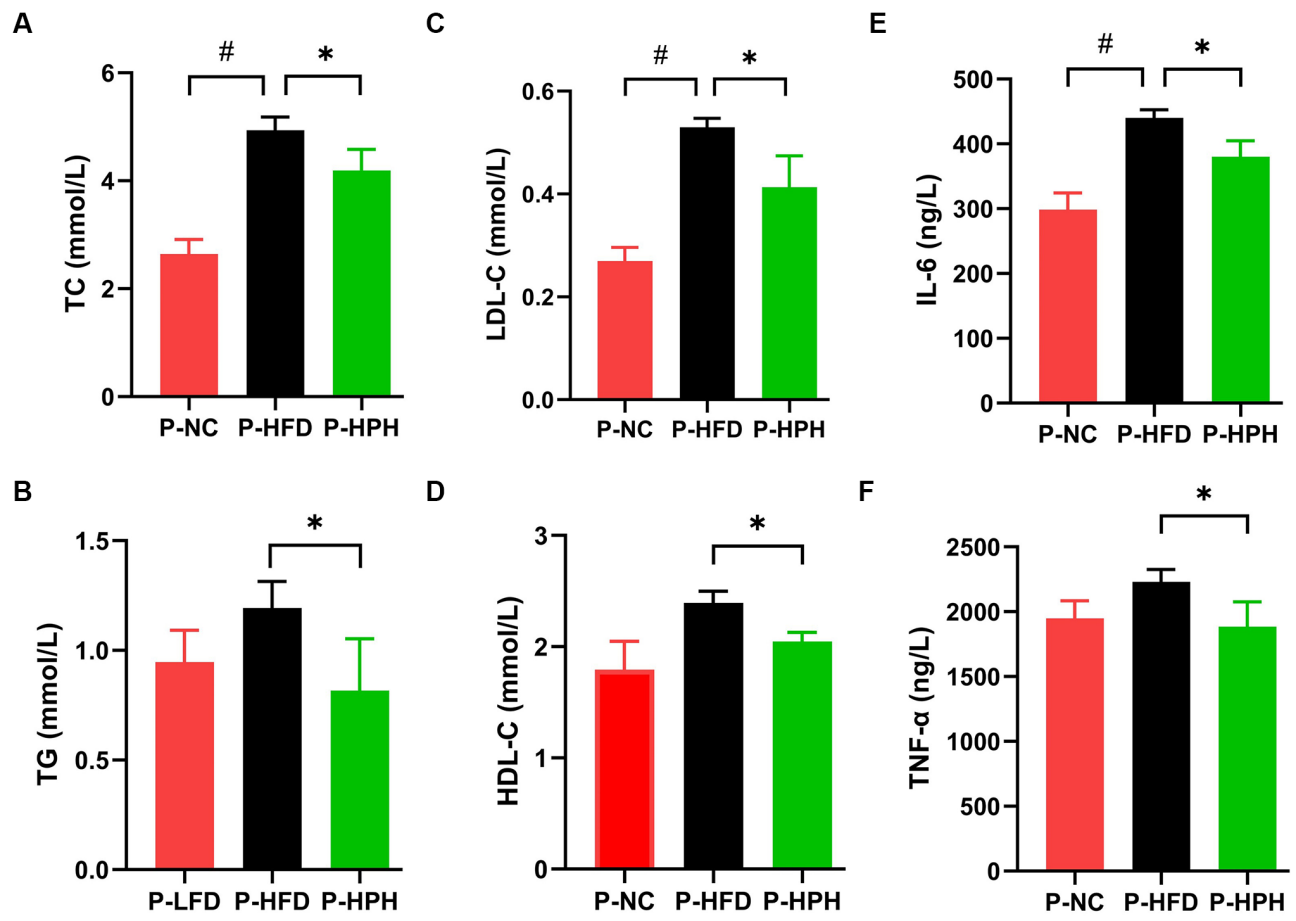
Effect of RLE in the prevention group on lipid and inflammatory factor levels in mice fed a high-fat diet. **(A–F)** Serum levels of TC, TG, LDL-C, HDL-C, IL-6, and TNF-α in mice. ^#^*p* < 0.05, P-HFD compared with P-LFD group. *p < 0.05, P-HFD compared with P-HPH group.

**Figure 4:**
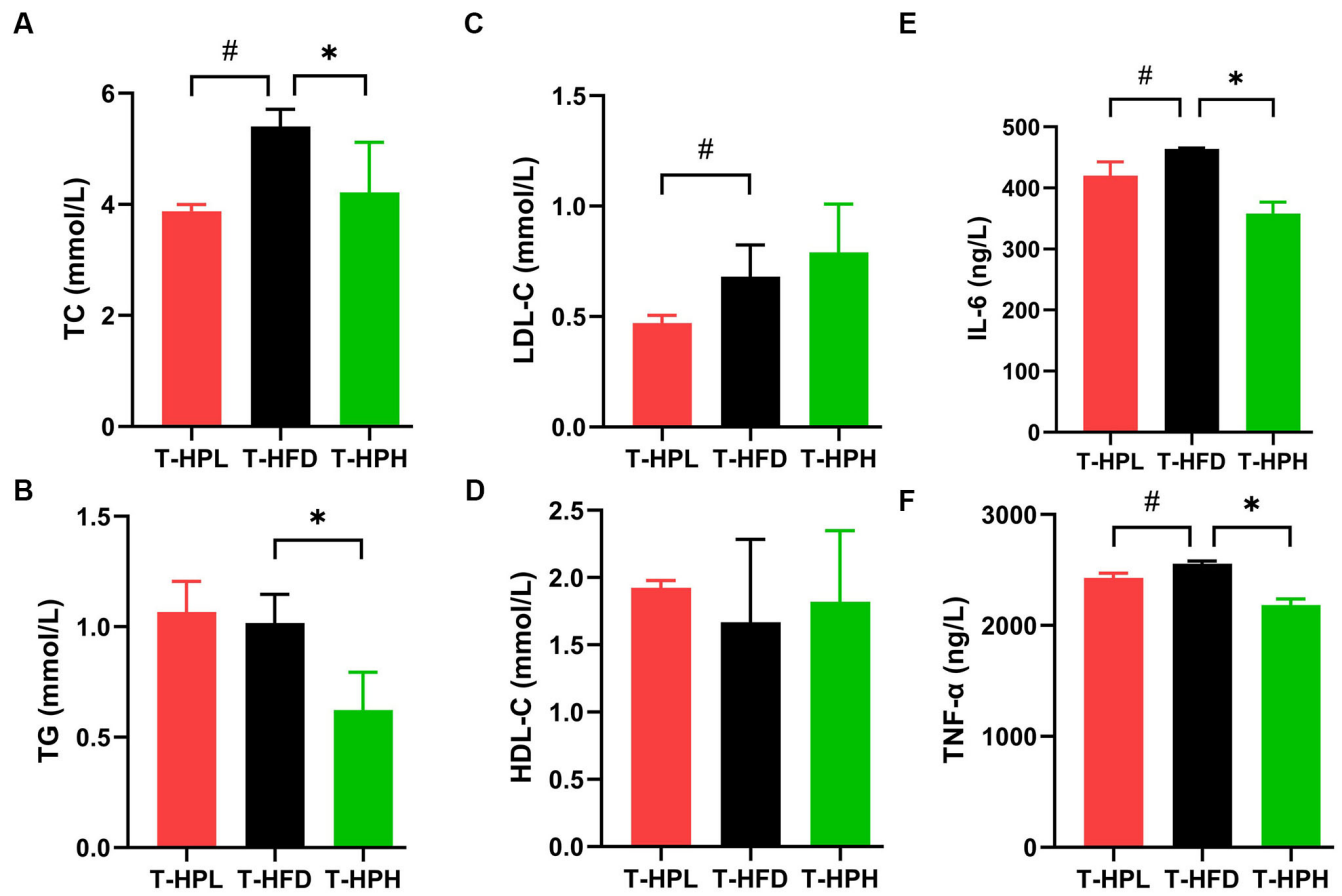
Effect of RLE in the treatment group on lipid and inflammatory factor levels in mice fed a high-fat diet. **(A–F)** Serum levels of TC, TG, LDL-C, HDL-C, IL-6, and TNF-α in mice. ^#^*p* < 0.05, T-HFD compared with T-HPL group. ^*^p < 0.05, T-HFD compared with T-HPH group.

The ameliorative effect of RLE on lipid accumulation induced by HFD was determined by measuring biochemical parameters, TC, TG, HDL-C, and LDL-C levels (Figures 3A–D). In the prevention stage, RLE reduced the HFD-induced increased in TC, TG, and LDL-C levels (Figures 4A–D). In addition, supplementing with RLE reduced the increase in IL-6 and TNF-α levels caused by HFD (Figures 3E,F). In the treatment stage, low-dose RLE significantly decreased TC and LDL-C levels, while high-dose RLE significantly decreased TG and TC levels. Both doses of RLE reduced IL-6 and TNF-α levels during the treatment stage (Figures 4E,F).

### Effects of RLE white adipose tissue and liver tissue

**Figure 5:**
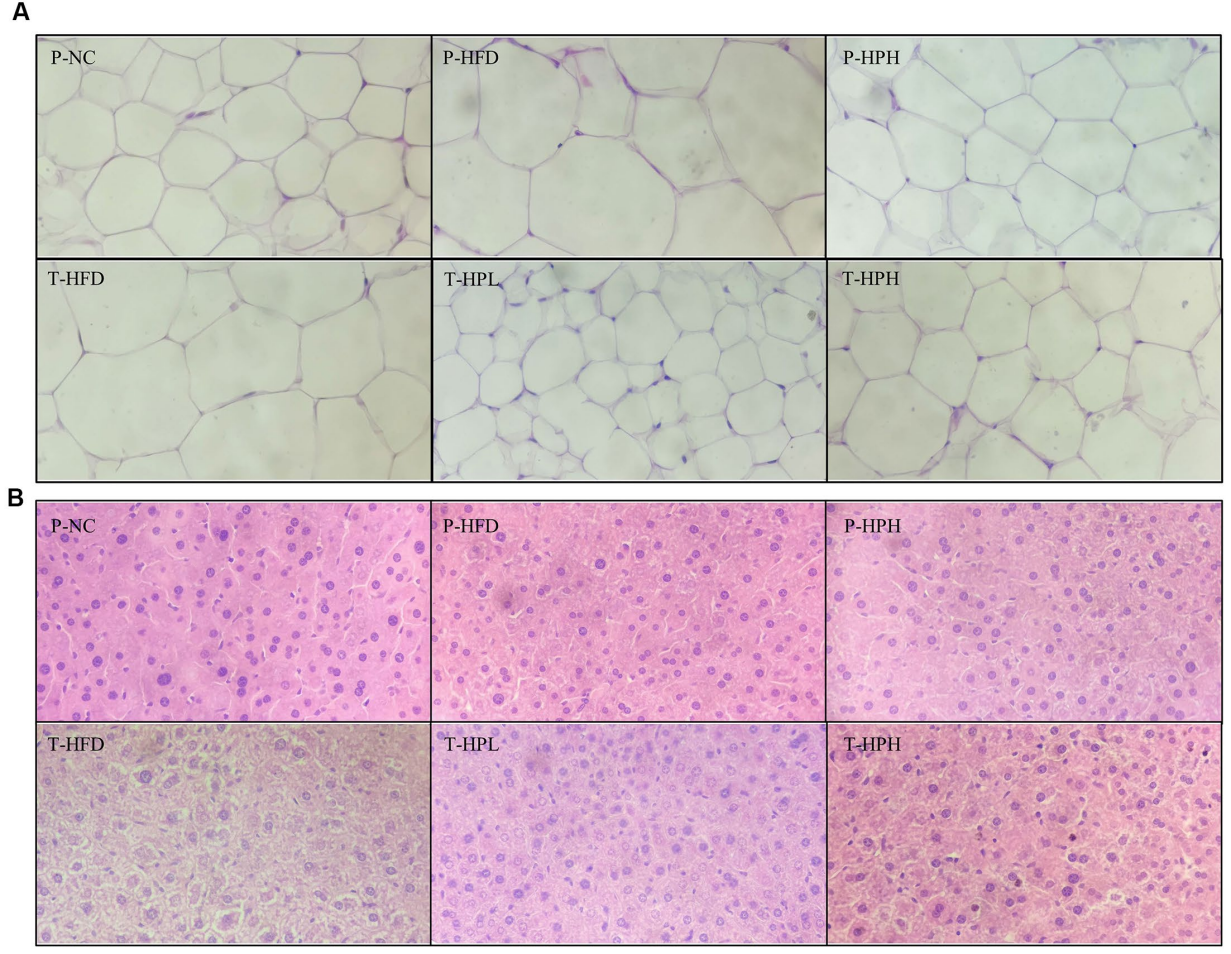
Histological observation of white adipose tissue and H&E staining of liver tissue in the prevention and treatment groups (400× magnification). **(A)** White adipose tissue. **(B)** Liver tissue.

In this study, white adipocytes were observed by H&E staining, and their sizes between groups were quantified using ImageJ software at the same scale. As shown in Figure 5A and Supplementary Table S5, the size of the P-HFD group significantly increased by about twice compared with the P-NC group, and the hypertrophic symptom was significantly alleviated in the P-HPH, T-HPL, and T-HPH groups with RLE supplementation. No significant liver fat accumulation was observed in all groups during the preventive stage, while during the treatment stage, T-HFD group continued to be fed HFD and showed significant differences in hepatocyte morphology and size, as well as noticeable lipid accumulation (Figure 5B).

### Effect of RLE supplementation on intestinal flora

As shown in Table 2, there was no significant difference in abundance and diversity between the P-HFD group and the P-NC group at the prevention stage (16 weeks) (*p* > 0.05), while the diversity in the P-HPH group was higher than both P-HFD and P-NC groups (*p* < 0.05). Similarly, the abundance and diversity indices of the T-HPL group significantly increased compared with the T-HFD group and T-HPH group (*p* < 0.05). Overall, P-HPH, T-HPL and T-HPH groups showed increases in diversity.

**Table 2:** Supplementary RLE effect on alpha diversity.

Groups	ACE	Chao	Shannon	Simpson
P-NC	555.85 ± 36.19^b^	555.59 ± 36.29^b^	6.09 ± 0.10^d^	0.019 ± 0.000^e^
P-HFD	575.70 ± 19.73^ab^	575.56 ± 19.71^ab^	6.19 ± 0.08^d^	0.017 ± 0.000^e^
P-HPH	503.21 ± 8.70^c^	503.08 ± 8.73^c^	6.89 ± 0.04^b^	0.088 ± 0.004^c^
T-HFD	591.59 ± 19.48^ab^	591.34 ± 19.50^ab^	6.68 ± 0.08^c^	0.029 ± 0.002^d^
T-HPL	604.73 ± 29.87^a^	604.72 ± 29.90^a^	7.66 ± 0.02^a^	0.156 ± 0.007^a^
T-HPH	513.24 ± 10.28^c^	513.02 ± 10.18^c^	6.93 ± 0.01^b^	0.106 ± 0.003^b^

Different letters were significantly different at the level of *p* < 0.05.

**Figure 6:**
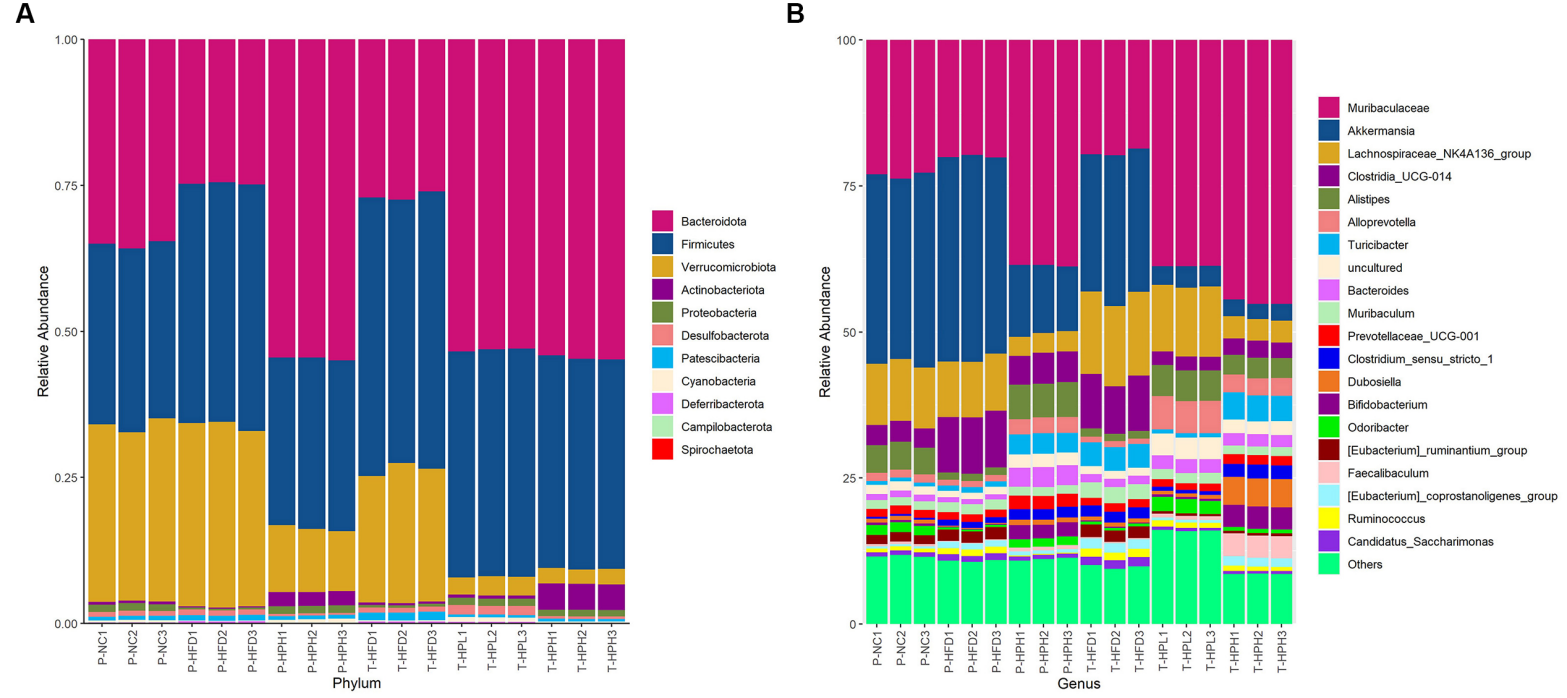
Effect of RLE on the composition of intestinal flora in HFD-induced obese mice. **(A)** Histogram of relative abundance at phylum level. **(B)** Histogram of relative abundance at genus level.

The composition and abundance of the intestinal microbiota in prevention and treatment groups were showed in Figure 6. *Bacteroidota* and *Firmicutes* were the most abundant phylum among 11 bacterial phyla displayed in each group. Compared with the P-NC group, the abundance of *Firmicutes* increased and the abundance of *Bacteroidota* decreased in P-HFD and T-HFD groups, indicating a higher *F*/*B* ratio. However, RLE supplementation increased the abundance of *Bacteroidota* and decreased the abundance of *Verrucomicrobiota*.

At the genus level, among the top 20 species, *Muribaculaceae*, *Akkermansia*, *Lachnospiraceae_NK4A136_group*, *Clostridia_UCG-014*, *Alistipes*, *Alloprevotella* and *Turicibacter* were the most abundant species. Compared with the corresponding HFD groups, the relative abundance of *Muriaculaceae*, *Alistipes* and *Alloprevotella* increased in all three groups supplemented with RLE. *Muriaculaceae* had the highest relative abundance (P-HPH: 38.6%, T-HPL: 38.7%, and T-HPH: 44.9%), while the relative abundance of *Akkermansia* and *Clostridia_UCG-014* decreased.

### Targets of polyphenols and genes by network pharmacology

**Figure 7:**
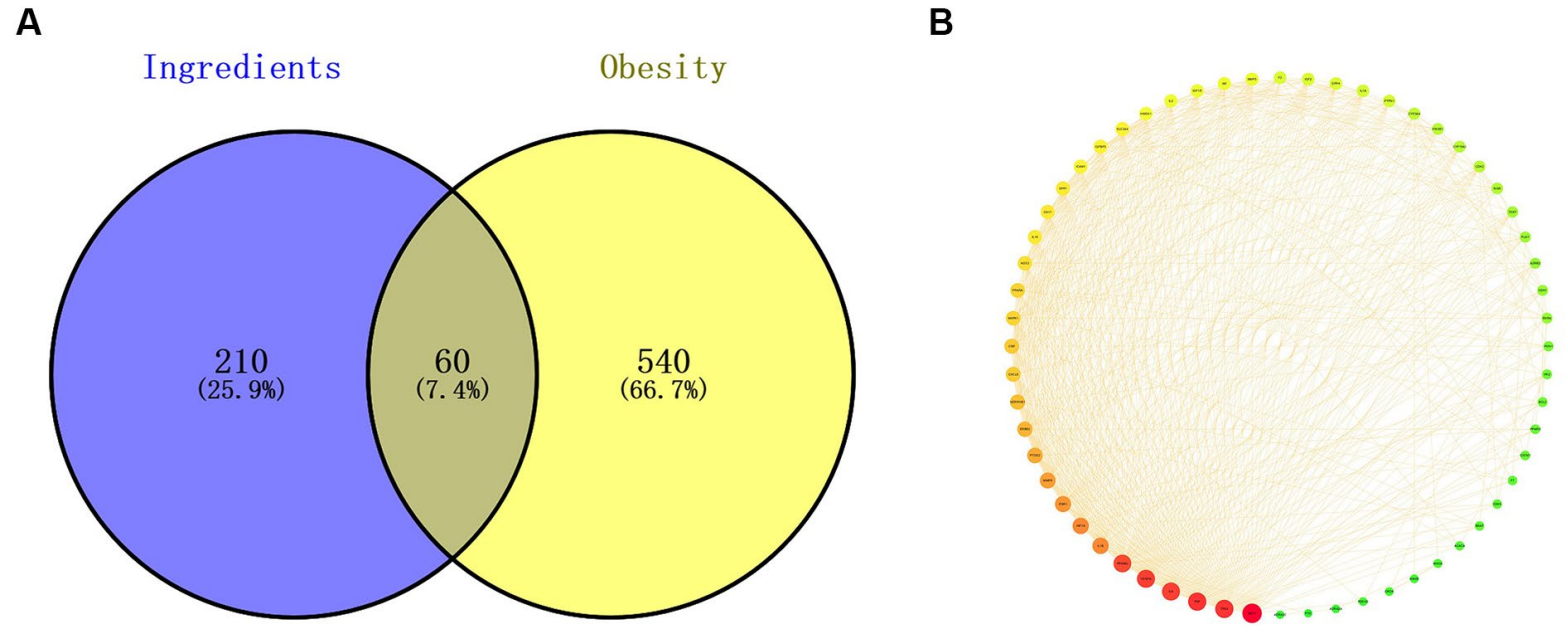
Overlapping targets and PPI network. **(A)** 60 overlapping targets of 7 polyphenols in RLE and obesity. **(B)** PPI network of 7 polyphenols in RLE and 60 overlapping targets of obesity.

Based on multiple studies and our results, polyphenols play a critical role in alleviating obesity from different perspectives. Therefore, weight control mechanisms of 7 typical polyphenols (chlorogenic acid, brevifolin carboxylic acid, ellagic acid, quercetin-3-O-rutinoside, Q3G, K3R, and quercetin) in RLE were explored through network pharmacology, especially the correlation between the weight control effect of RLE and these polyphenols. For polyphenol-related targets, a total of 160 and 269 potential targets were screened out from the TCMSP and Swiss Target Predictiondatabases, respectively. After combining the two databases and removing duplicate target, 270 potential targets for 7 RLE polyphenols were obtained (Supplementary Table S1). For obesity-related targets, a total of 600 potential targets were identified after removing duplicates from the GeneCards, OMIM, and TTD databases, among which those with eligible relevance scores >5 were recognized as potential targets in the GeneCards database (Supplementary Table S2). Then, the overlapping targets of the seven polyphenols in RLE and obesity were visualized by Veen diagram (Figure 7A). Finally, a total of 60 overlapping targets were obtained as potential therapeutic targets of 7 polyphenols in RLE for the treatment of obesity.

### PPI networks and key target genes

The common 60 targets of 7 polyphenols and obesity were imported into the STRING database to construct a PPI network (Figure 7B), which had 60 nodes and 607 edges. The larger the degree value, the larger size and darker red color of the nodes, indicating a higher probability of being a target of polyphenols in RLE for obesity treatment. The PPI network was downloaded and uploaded to Cytoscape 3.9.0 software, the three parameters “BC,” “CC,” and “DC” were calculated, and the middle values of the three parameters were used as the filtering condition. The three thresholds were “BC” > 51.933, “CC” > 0.009, and “DC” > 20.233, respectively. Nodes that meet these three thresholds were considered as key target. A total of 12 potential key targets for treating obesity with RLE polyphenols were AKT1, TP53, TNF, IL-6, VEGFA, PPARG, HIF1A, ESR1, ERBB2, CRP, MAPK1, and PPARA.

### Go and KEGG enrichment analysis

**Figure 8:**
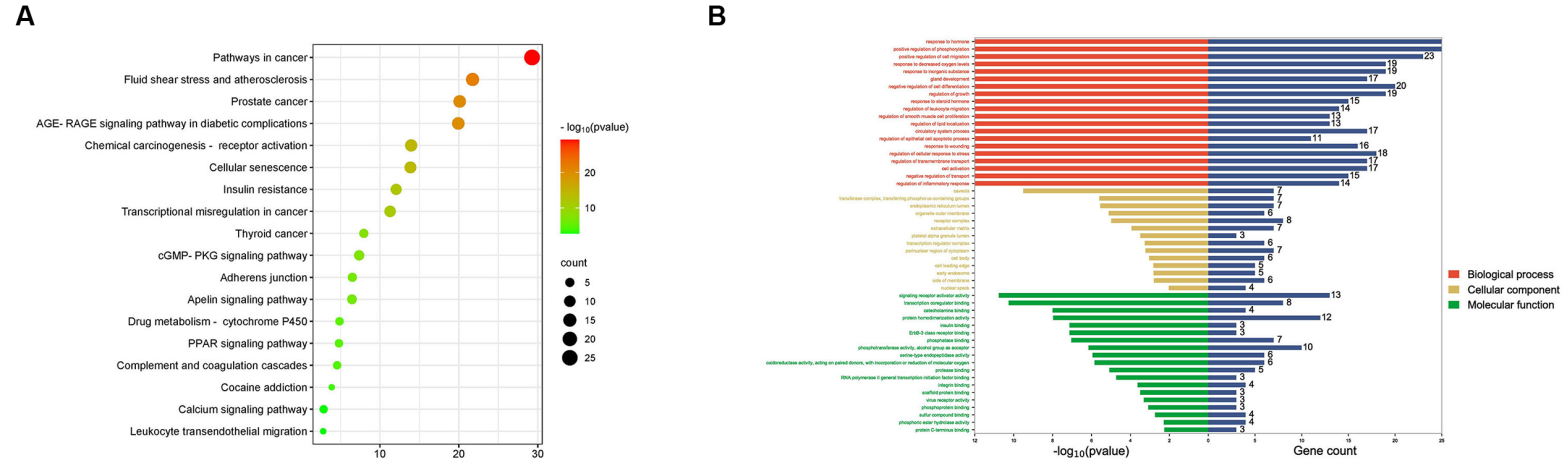
GO and KEGG enrichment analysis. **(A)** GO pathway enrichment analysis. **(B)** KEGG pathway enrichment analysis.

The sixty overlapping targets were uploaded to the Metascape database using *Homo sapiens* (species) for GO and KEGG enrichment analysis. A total of 53 items were obtained from the GO enrichment, all with *p* < 0.05 (Supplementary Table S3). BP, CC, and MF were 20, 14 and 19 items, respectively, and then BP, CC and MF genes were visualized (Figure 8B). The results showed that hormone to response, concave and signaling receptor activator activity were the most significant. Moreover, KEGG enrichment analysis yielded 18 signaling pathways with *p* < 0.05 (Supplementary Table S4), and they were plotted by bubble maps for visualization and analysis (Figure 8A). The results showed that the major pathways included cancer pathways, fluid shear stress and atherosclerosis, and prostate cancer.

## Discussion

Obesity is one of the major public health problems worldwide, and long-term intake of HFD can lead to fat accumulation, metabolic disorders, and an increased risk of obesity (3). Compared to drugs that can produce certain side effects, dietary intervention with natural diets rich in polyphenols can be a promising means of treating obesity. These bioactive compounds in dietary diets can act as antioxidants and anti-inflammatory agents, which is beneficial for weight loss and alleviation of metabolic disorders (7). Raspberry leaf, a by-products with huge yield (> 1 million tons/per year) has high health care and medicinal values in our previous studies (13, 14). It has been reported that raspberry leaf containing quercetin, ellagic acid, chlorogenic acid, quercetin-3-O-rutinoside, and Q3G polyphenols exhibit good antioxidant activity (19, 47). In this study, the TPC of the RLE was more than 50% and the most important phenolic compounds of RLE were Q3G, K3R and ellagic acid. RLE significantly reduced the body weight of HFD-induced obese mice in both prevention and treatment phases, but the obesity-relieving effects of RLE and possible underlying mechanisms remain limited.

Obesity also is recognized as a chronic low-grade inflammation. Higher levels of IL-1β and IL-6 are characteristics of chronic low-grade inflammation (26). Studies have shown that polyphenols and extracts rich in polyphenols have beneficial effects on obesity-related inflammation. This is not only related to high polyphenol content, but also involves the synergistic effects between polyphenol molecules in regulating obesity and related complications (7). For example, Q3G inhibits the production of IL-6 and TNF-α (48), as well as quercetin inhibits the inflammatory factors IL-1β and IL-6, and stimulates the secretion of the anti-inflammatory factor (49). Quercetin-3-O-rutinoside reduces glucose uptake by modulating the PPAR-γ signaling pathway (50). Chlorogenic acid also weakens disordered lipid metabolism through PPAR-α (51). In the study of obesity-related signaling pathways, AKT regulated energy homeostasis by maintaining the level of ATP in cells and initiated lipolysis (52), while PPAR was found to be an adipose gene sensor with fat-burning, lipid metabolism and inflammatory response effects (53). This suggests that the significant reduction of IL-6 and TNF-α inflammation levels in HFD-induced obese mice by RLE may be due to its abundance of polyphenolic substances. In this study, although the interaction analysis of the 7 main phenolic compounds in RLE and obesity through network pharmacology had predicted 12 potential targets, based on experimental results and literature reports, it was speculated that IL-6, TNF, AKT1, and PPAR may be key targets for RLE polyphenols in regulating obesity.

The composition and function of the intestinal flora are thought to be associated with obesity, which can be alleviated by regulating the intestinal flora (54). In this study, diversity is increased and increase the *F/B* ratio in both RLE-supplemented groups compared with the corresponding HFD group. This indicate that RLE supplementation has improved the diversity of intestinal flora in obese mice during prevention and treatment stages. At phylum level, *Verrucomicrobiota* abundance decreased in all three groups supplemented with RLE. Study shows that the intestinal flora of patients with inflammatory bowel disease (IBD) reveals high levels of *Verrucomicrobiota* in the feces of IBD patients (55). At the genus level, RLE supplementation can up-regulate *Muriaculaceae*, *Alistipes* and *Alloprevotella* abundance, while the relative abundance of Akkermansia and Clostridia_UCG-014 decreased. Genomic analysis reveal that *Muribaculaceae* is functionally diverse in the degradation of complex carbohydrates, and this species has also been implicated in the regulation of obesity, with low abundance in obesity models (25, 56), this is consistent with our findings. It has been shown that *Muriaculaceae*, *Alistipes* and *Alloprevotella* are associated with colitis and are negatively correlated with inflammation (57). *Lachnospiracea_ NK4A136_ Group*, *Alistipes*, and *Alloprevotella* have been reported to be producers of SCFAs, which plays an active role in the prevention and treatment of obesity (58, 59). It is worth mentioning that *Akkermansia muciniphila* is generally considered a beneficial bacterium and it can reduce fat storage and alleviate lipid metabolism disorders (60). In animal models, administration of polyphenols, fructo oligosaccharides, oat bran, and whole grain barley increased the abundance of *Akkermansia*, which proved to be lower in mice fed HFD and all had obesity or type II diabetes-like symptoms. However, opposite results were found in some rat studies, an increase in the abundance of *Akkermansia* was observed in HFD rats supplemented with barley, while a decrease in the abundance of *Akkermansia* was observed in NC diet supplemented with barley. These were consistent with our experimental results. The different result may be the result of environmental and genetic factors (61). In addition, Clostridia_UCG-014 abundance was higher in the obesity model. Overall, RLE can alleviate HFD-induced obesity by improving intestinal flora.

## Conclusion

In this study, the effects of RLE supplementation on HFD-induced obese mice carefully evaluated. During the prevention and treatment stages, RLE alleviated weight gain, fat accumulation, lipid disorders, inflammatory levels, and improved intestinal flora in HFD-induced obese mice. The potential mechanism may be attributed to the specific polyphenols of RLE and their synergistic effects on IL-6, TNF, AKT1 and PPAR pathways. RLE has a great anti-obesity potential and may be used as a means to alleviate obesity and related diseases. It may also promote the application of raspberry leaves or raspberry leaf extracts, and the development of related products.

## Data availability statement

The datasets presented in this study can be found in online repositories. This data can be found here: Sequence Read Archive (SRA) database is BioProject ID: PRJNA1025257 (https://www.ncbi.nlm.nih.gov/bioproject/?term=PRJNA1025257).

## Ethics statement

The animal study was approved by Committee on Biological and Medical Ethics, North University of China. The study was conducted in accordance with the local legislation and institutional requirements.

## Author contributions

TW: Data curation, Formal analysis, Investigation, Software, Visualization, Writing – original draft. JY: Funding acquisition, Methodology, Supervision, Validation, Visualization, Writing – review & editing. ZH: Data curation, Formal analysis, Validation, Writing – original draft. FW: Data curation, Visualization, Writing – original draft. RL: Data curation, Investigation, Writing – original draft. YL: Data curation, Formal analysis, Writing – review & editing. XL: Data curation, Writing – review & editing.

## Footnotes

^1^ https://old.tcmsp-e.com/tcmsp.php

^2^ http://swisstargetprediction.ch/

^3^ https://www.uniprot.org/

^4^ https://pubchem.ncbi.nlm.nih.gov/

^5^ https://www.genecards.org/

^6^ https://www.omim.org/

^7^ https://db.idrblab.net/ttd/

^8^ http://jvenn.toulouse.inra.fr/app/index.html

^9^ https://cn.string-db.org/

^10^ https://metascape.org

^11^ https://www.bioinformatics.com.cn/
