# Supplementary material for: Integrated 16s RNA sequencing and network pharmacology to explore the effects of polyphenol-rich raspberry leaf extract on weight control
Source: Front Nutr. 2024 Jan 8;10:1306037. doi: 10.3389/fnut.2023.1306037 (PMC10800909; doi:10.3389/fnut.2023.1306037)
Supplement: Supplementary file 2 [file Data_Sheet_2.docx]

Table S5

Adipocyte sizes in prevention group and in treatment group.

| Group | Area (μm^2^) | Group | Area (μm^2^) |
| --- | --- | --- | --- |
| P-NC | 2334.31±757.48c | T-HPL | 2139.28±605.44c |
| P-HFD | 5097.31±975.35a | T-HFD | 4964.10±841.72a |
| P-HPH | 3866.71±1042.90b | T-HPH | 4172.09±568.79b |

Different letters were significantly different at the level of *p* < 0.05.
